# Supplementary material for: Plasma Vitamin C Levels: Risk Factors for Deficiency and Association with Self-Reported Functional Health in the European Prospective Investigation into Cancer-Norfolk
Source: Nutrients. 2019 Jul 9;11(7):1552. doi: 10.3390/nu11071552 (PMC6682997; doi:10.3390/nu11071552)
Supplement: Supplementary file 1 [file nutrients-11-01552-s001.pdf]

Table S1. Characteristics of EPIC Norfolk in quartiles of plasma vitamin C level.

		1.0		2.0		3.0		4.0	
Quartile range (mmol/l)		<41.0		41 -53.9		54 – 65.9		≥66	
Age (mean SD)		60.4	(9.43)	59.1	(9.4)	58.4	(9.09)	58.8	(9.15)
Sex	Male	3,536	(63.8)	2,933	(53.8)	2,306	(40.3)	1,492	(25.9)
	Female	2,007	(36.2)	2,520	(46.2)	3,417	(59.7)	4,263	(74.1)
BMI (Median IQR)		26.5	(24.3-29.0)	26.3	(24.1-28.79)	25.6	(23.6-28.1)	25.0	(23.0-27.3)
Physical functioning	Mean (SD)	74.6	(24.9)	78.5	(22.2)	81.4	(20.2)	80.8	(20.87)
Role limitation due to physical problem	Mean (SD)	70.2	(40.12)	75.0	(37.4)	78.2	(35.5)	77.7	(35.7)
Bodily pain	Mean (SD)	72.2	(24.96)	73.9	(23.01)	75.5	(22.5)	75.1	(22.4)
Vitality	Mean (SD)	59.6	(21)	61.1	(20)	62.1	(19.3)	62.6	(19.5)
General health	Mean (SD)	66.0	(21.3)	69.0	(19.8)	71.2	(18.8)	71.8	(19.1)
Number (%)									
Smoking status	Current smoker	1155	(21)	552	(10.2)	445	(7.8)	403	(7.1)
	Former smoker	2483	(45.2)	2414	(44.5)	2331	(41.1)	2233	(39.1)
	Non-smoker	1857	(33.8)	2455	(45.3)	2897	(51.1)	3076	(53.9)
Physical activity	Inactive	2101	(37.9)	1638	(30)	1576	(27.5)	1466	(25.5)
	Moderately inactive	1423	(25.7)	1560	(28.6)	1695	(29.6)	1742	(30.3)
	Moderately active	1145	(20.7)	1248	(22.9)	1337	(23.4)	1398	(24.3)
	Active	874	(15.8)	1006	(18.5)	1115	(19.5)	1149	(20)
Alcohol intake	None	1196	(22.5)	986	(18.7)	1067	(19.2)	1014	(18.1)
	1 - ≤7 units a week	2433	(45.7)	2409	(45.6)	2560	(46.1)	2619	(46.8)
	>7 - ≤14 units a week	884	(16.6)	1073	(20.3)	1136	(20.5)	1121	(20)
	>14 units a week	812	(15.2)	811	(15.4)	791	(14.2)	843	(15.1)
Townsend score	Least deprived (<−3.82)	980	(17.7)	1042	(19.2)	1216	(21.3)	1210	(21.1)
	2 (−3.82 to −2.96)	1023	(18.5)	1119	(20.6)	1176	(20.6)	1175	(20.5)
	3 (−2.96 to − 2.16)	1073	(19.4)	1111	(20.5)	1159	(20.3)	1103	(19.3)
	4 (−2.16 to −0.74)	1141	(20.6)	1129	(20.8)	1089	(19.1)	1127	(19.7)
	Most deprived (>−0.74)	1309	(23.7)	1026	(18.9)	1063	(18.6)	1113	(19.4)
Education below	Lower than O level	2921	(52.7)	2561	(47)	2492	(43.6)	2563	(44.5)

	A-level or above	2621	(47.3)	2887	(53)	3227	(56.4)	3191	(55.5)
Self-reported - Prevalent illness	Cerebrovascular incident	122	(2.2)	74	(1.4)	54	(0.9)	60	(1)
	Myocardial infarction	275	(5)	179	(3.3)	141	(2.5)	101	(1.8)
	Cancer	297	(5.4)	268	(4.9)	272	(4.8)	364	(6.3)
	Diabetes mellitus	203	(3.7)	148	(2.7)	95	(1.7)	58	(1)
	Asthma	449	(8.1)	464	(8.5)	479	(8.4)	501	(8.7)
Vitamin C supplementation	No supplementation	4011	(72.7)	3377	(62.3)	3141	(55.2)	2420	(42.2)
	Taking supplementation	1506	(27.3)	2046	(37.7)	2548	(44.8)	3313	(57.8)
Social class	Non-manual occupations	2740	(50.9)	3159	(59.1)	3591	(64)	3768	(66.7)
	Manual occupations	2648	(49.1)	2190	(40.9)	2020	(36)	1882	(33.3)
BMI	<25 kg/m2	1795	(32.4)	1852	(34)	2387	(41.7)	2864	(49.8)
	25-<30 kg/m2	2689	(48.5)	2659	(48.8)	2581	(45.1)	2340	(40.7)
	≥30 kg/m2	1059	(19.1)	942	(17.3)	755	(13.2)	551	(9.6)

Table S2. Linear regression models for the association between vitamin C and self-reported health.

			Physical function		Role physical health		Bodily Pain		Vitality		General health	
			Beta	95% CI	Beta	95% CI	Beta	95% CI	Beta	95% CI	Beta	95% CI
1 SD change in vitamin C			1.35	(1.01-1.68)	1.68	(1.07-2.30)	0.65	(0.26-1.03)	0.99	(0.66-1.33)	1.04	(0.71-1.37)
	With supplementation		1.15	(0.66-1.65)	0.57	(-0.34-1.49)	0.57	(0.0048-1.13)	0.74	(0.26-1.22)	0.59	(0.12-1.07)
	Without supplementation		1.77	(1.29-2.25)	3.5	(2.63-4.37)	1.42	(0.87-1.98)	1.55	(1.07-2.04)	1.73	(1.25-2.20)
Spline linear regression models in those without supplementation	Vitamin C level mmol/L	>0	0.16	(0.094-0.23)	0.26	(0.14-0.38)	0.11	(0.027-0.18)	0.1	(0.031-0.17)	0.12	(0.049-0.18)
		≥40	-0.088	(-0.21-0.034)	-0.13	(-0.35-0.092)	-0.036	(-0.18-0.11)	-0.029	(-0.15-0.095)	-0.01	(-0.13-0.11)
		≥60	-0.058	(-0.18-0.062)	-0.0037	(-0.22-0.21)	-0.052	(-0.19-0.088)	-0.02	(-0.14-0.10)	-0.095	(-0.21-0.024)
		≥110	0.37	(-0.45-1.18)	0.27	(-1.21-1.75)	0.98	(0.015-1.94)	0.4	(-0.45-1.26)	0.59	(-0.22-1.40)

* Spline model. All models adjusted for age, sex, smoking, physical activity, alcohol intake, BMI, education status, Townsend index and previous medical history (Cancer, diabetes, MI, Asthma and CVA). All outcomes compared the bottom decile vs. all other deciles except role physical health, which was defined the comparison as the bottom quintile vs. re
